# Meet the IUPAB Councilor—Hans-Joachim Galla

**DOI:** 10.1007/s12551-021-00879-6

**Published:** 2021-11-23

**Authors:** Hans-Joachim Galla

**Affiliations:** Institute for Biochemistry, Westfälische Wilhelms Universität Muenster, Corrensstr. 36, 48149 Münster, Germany

## Abstract

As one of the twelve Councilors, it is my pleasure to provide a short biographical sketch for the readers of *Biophys. Rev*. and for the members of the Biophysical Societies. I have been a member of the council in the former election period. Moreover, I served since decades in the German Biophysical Society (DGfB) as board member, secretary, vice president, and president. I hold a diploma degree in chemistry as well as PhD from the University of Göttingen. The experimental work for both qualifications has been performed at the Max Planck Institute for Biophysical Chemistry in Göttingen under the guidance of Erich Sackmann and the late Herman Träuble. When E. Sackmann moved to the University of Ulm, I joined his group as a research assistant performing my independent research on structure and dynamics of biological and artificial membranes and qualified for the “habilitation” thesis in Biophysical Chemistry. I have spent a research year at Stanford University supported by the Deutsche Forschungsgemeinschaft (DFG) and after coming back to Germany, I was appointed as a Heisenberg Fellow by the DFG and became Professor in Biophysical Chemistry in the Chemistry Department of the University of Darmstadt. Since 1990, I spent my career at the Institute for Biochemistry of the University of Muenster as full Professor and Director of the institute. I have trained numerous undergraduate, 150 graduate, and postdoctoral students from chemistry, physics, and also pharmacy as well as biology resulting in more than 350 published papers including reviews and book articles in excellent collaboration with colleagues from different academic disciplines in our university and also internationally, e.g., as a guest professor at the Chemistry Department of the Chinese Academy of Science in Beijing.


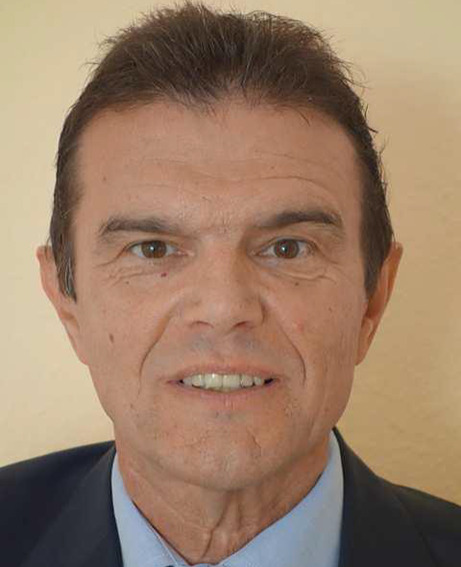


## Contributions to biophysics

My research involves experimental approaches to investigate the structural and functional organization of biological and reconstituted lipid bilayer membranes starting in the mid-70 s with the spectroscopical determination of the lipid and protein lateral diffusion (Galla and Sackmann 1974; Kapitza et al. 1984) and the lateral organization of membranes containing mixed lipid composition including negatively charged lipids. My major focus was the lateral membrane organization and chemically induced phase separation phenomena induced by Ca2 + (Galla and Sackmann 1975) or simple polypeptides (Hartmann and Galla 1977). This work later on was expanded to the membrane interaction of small biological peptides like melittin (Steinem et al. 1998) and seminalplasmin (Galla et al. 1985) or viral peptides (Hinz and Galla 2005) or the positively charged peptide antibiotic polymyxin (Hartmann et al. 1978; Sixl and Galla 1979). Domain formation of the real membrane proteins glycophorin (Tampé et al. 1989) and annexin (Drücker et al. 2014; Grill et al. 2018) is investigated as well. In Stanford I investigated the effect of high pressure and anesthetics on membrane fluidity and phase separation (Galla and Trudell 1980). Different techniques like spin label electron paramagnetic resonance, fluorescence after photobleaching (FRAP), excimer technique, ad fluorescence polarization were applied.

Besides the organization of bilayer structures, we investigated lipid and lipid-peptide monolayers by film balance experiments including fluorescence and Brewster angle microscopy and transfer techniques to allow atomic force microscopic investigations of the monolayer structure. The biological relevant systems under biophysical investigation were lung surfactants (Post et al. 1995; Krol et al. 2000) and tear films (Dwivedi et al., 2014a, b) including the effect of nanoparticles on the structure of these monolayer films (Harishchandra et al. 2010, Sachan et al. 2012). For the first time, TOF–SIMS analysis was used to image the structure and the chemical composition of the domains formed within the monolayer (Breitenstein et al. 2006). The 3D structure of the lung surfactant was visualized by atomic force microscopy using air bubbles coated with the surfactant in order to come closer to the natural system with a curved surface (Knebel et al. 2002).

In the last years, our focus in collaboration with F. Glorius from our university moved to the effect of imidazolium containing ionic liquids on membrane structures (Wang et al. 2015, 2016, 2018). This new topic was also introduced into the programs of the recent EBSA and IUPAB meetings (Benedetto and Galla 2018).

Besides this topic of membrane biophysics, we had a focus on transport processes across cellular barrier which in our hands was the blood–brain barrier (BBB). We developed cell culture models of cerebral endothelial cells also in co-culture with other cells relevant to the cerebrovascular unit (Franke et al. 1999; Angelow et al. 2004). These cell culture models were used to investigate the structural organization of the cellular contacts called tight junctions (Hein et al., 1992a, b, Grebenkämper and Galla 1994) and the passive as well as active transfer of substrates of pharmacological interest across this barrier between the blood and the brain (Grapp et al. 2013) including the use of nanoparticles (Qiao et al. 2012, Rempe et al. 2014). A new technique, the impedance spectroscopy, across cellular barriers was developed to allow the quantification of the barrier tightness (Erben et al. 1995; Wegener et al. 1996; Benson et al. 2013) and correspondingly the modulation of the barrier with the aim to open the blood–brain barrier temporarily to allow a transfer of pharmaceutical compounds to the otherwise restricted access to the brain. This is an important step to treat brain diseases including also the application of nanoparticles as vehicles to cross the BBB. The impedance technique has been brought to the commercial market in collaboration with the Nanoanalytics Company at the Center for Nanotechnology in Muenster (https://www.nanoanalytics.com/de/produkte/cellZscope.html).
